# Does a Gluten-Free Diet Improve Quality of Life and Sleep in Patients with Non-Coeliac Gluten/Wheat Sensitivity?

**DOI:** 10.3390/nu15153461

**Published:** 2023-08-04

**Authors:** Connor Cotton, Suneil A. Raju, Hamza Ahmed, Grace Webster, Rachel Hallam, Iain Croall, Sarah Coleman, Nick Trott, Anupam Rej, Mohamed G. Shiha, Imran Aziz, David S. Sanders

**Affiliations:** 1 Academic Unit of Gastroenterology, Sheffield Teaching Hospitals, Sheffield S10 2JF, UK; suneil.raju@nhs.net (S.A.R.); grace.webster1@nhs.net (G.W.); shcoleman33@gmail.com (S.C.); nick.trott@nhs.net (N.T.); anupam.rej@nhs.net (A.R.); imran.aziz1@nhs.net (I.A.); david.sanders1@nhs.net (D.S.S.); 2 Department of Infection, Immunity and Cardiovascular Disease, The University of Sheffield, Sheffield S10 2TN, UK; 3 Academic Unit of Radiology, University of Sheffield, Sheffield S10 2TN, UK

**Keywords:** Non-Coeliac Gluten/Wheat Sensitivity, NCGWS, Coeliac Disease, gluten-free diet, quality of life, sleep

## Abstract

Introduction: The role of a gluten-free diet (GFD) in Non-Coeliac Gluten/Wheat Sensitivity (NCGWS) is unclear. We present the largest study comparing adherence to a GFD in patients with Coeliac Disease (CD) and NCGWS and assess its impact on quality of life (QoL) and sleep in patients with NCGWS. Methods: Patients with NCGWS at a tertiary centre completed the Coeliac Disease Adherence Test (CDAT), Coeliac Symptom Index (CSI) and Sleep Condition Indicator (SCI). Higher CDAT scores indicate worse adherence, higher CSI scores indicate poorer QoL, and higher SCI scores indicate better sleep. CDAT scores were correlated with CSI and SCI scores. A second group of patients with CD completed the CDAT questionnaire only. Results were compared with the CDAT responses from the NCGWS group. Results: For the NCGWS cohort (*n* = 125), the median CDAT score was 17/35, indicating poor adherence. The median CSI score was 44/80, with 40% of scores associated with a poor QoL. The median SCI score was 14/32, and DSM-V criteria for insomnia was met by 42% of patients. There was a positive correlation between CSI and CDAT scores (r = 0.59, *p* < 0.0001) and a negative correlation between SCI and CDAT scores (r = −0.37, *p* = 0.0002). In the CD cohort (*n* = 170), the median CDAT score was 13/35. Patients with NCGWS had poorer adherence compared to CD (CDAT: 17.0 vs. 13.0, respectively, *p* = 0.0001). Conclusion: Patients with NCGWS adhere to a GFD less than those with CD. Poorer adherence to a GFD in patients with NCGWS correlates with a worse QoL and sleep performance.

## 1. Introduction

Coeliac Disease (CD) and Non-Coeliac Gluten/Wheat Sensitivity (NCGWS) are gluten-related disorders that can present with gastro-intestinal and extra-intestinal symptoms. The mainstay of treatment for both conditions is a gluten-free diet (GFD) [1].

CD is characterised by an immune-mediated gluten sensitive enteropathy triggered by the ingestion of gluten and affects 1% of the general population [1,2]. It is diagnosed using serological testing with endomysial (EMA) antibodies and tissue transglutaminase (TTG) antibodies. Individuals with positive serology should undergo a confirmatory gastroscopy with duodenal biopsy to confirm the presence of villous atrophy, the hallmark of CD [3]. NCGWS was first described in the late 1970s [4] and is characterised by a very similar presentation to CD but without the typical serological and histological changes seen in CD [1,4]. There appears to be a significant crossover in the symptoms displayed in NCGWS and Irritable Bowel Syndrome (IBS), with intestinal symptoms such as abdominal pain, diarrhoea and bloating being common [5]. Extra-intestinal symptoms include fatigue, myalgia, headache and brain fog [5,6]. Moreover, a relationship may exist between NCGWS and the development of neurological and psychiatric manifestations, such as depression, schizophrenia and ataxia [7]. Symptoms tend to develop within hours to a day following the ingestion of gluten [5]. 

There is currently no accepted biomarker for the diagnosis of NCGWS [8], which can make diagnosis more challenging. However, patients with NCGWS have been found to have significantly higher levels of IgG4 than patients with CD and healthy controls and higher IgG2 levels than healthy controls. On the other hand, patients with CD have been found to have elevated levels of IgG1 and IgG3 [8,9]. Furthermore, patients with NCGWS also have increased antibody reactivity to bacterial lipopolysaccharide and flagellin, as well as elevated levels of soluble CD14 and lipo-polysaccharide-binding protein [10]. This implies there is an alternative immune response in NCGWS compared to CD [8,9,10]. Histologically, there have been several markers indicative of NCGWS found. Carroccio et al. have shown that duodenal and rectal mucosal biopsies from patients with a diagnosis of NCGWS had a higher number of immune cells and eosinophils than tissues from controls. Eosinophil infiltration was greater in the rectal biopsies compared with duodenal biopsies. They therefore suggested that evaluation of patients with NCGWS should include rectal biopsies, and that infiltration of eosinophils in the absence of endoscopic findings could be a marker of NCGWS [11]. 

At present, The Salerno Expert’s Criteria is regarded as the gold-standard method for diagnosis of NCGWS, which first requires the exclusion of CD and IgE-mediated wheat allergy [1,4,7]. After a period of treatment with a GFD, patients should undergo a double-blind placebo-controlled gluten challenge followed by a one-week washout strict GFD. A positive diagnosis can be made if there is a 30% improvement in symptoms following initiation of a GFD and if there is a variation of at least 30% between the gluten and placebo challenge [7]. 

However, randomized clinical trials performing similar gluten challenges report a strong nocebo response [12]. The Salerno group recommends repeating the challenge to minimise this effect [7]. Nonetheless, this makes the diagnostic process for NCGWS challenging and time intensive. 

Interestingly, it has been found that not all gluten-containing foods cause the same degree of intolerances, and that gluten may not be the sole causal agent in perceived gluten intolerance. Compelling evidence for this comes from Jansson-Knodell et al., who collected data on over 2000 respondents looking at self-reported gluten sensitivity. They found the overall rate of gluten intolerance was 5.1%, but that each gluten-containing food had different rates of intolerances (wheat: 4.8%, flour: 1.2%, barley: 0.8% and rye: 0.8%), whilst only 0.3% reported an intolerance to all gluten-containing foods [13]. This suggests that wheat may be the main driver of intolerances and is why the term Non-Coeliac Gluten/Wheat Sensitivity is more often used rather than the previously used Non-Coeliac Gluten Sensitivity. Other estimates put the prevalence of NCGWS to be between 0.6 and 13% worldwide [1,14].

Whilst the only effective treatment for CD is a GFD, which can lead to symptom improvement within days to weeks [15,16], there is much less known about the duration and need for adherence to a GFD in patients with NCGWS [1]. It has been shown that patients with NCGWS do see an improvement in their symptoms following the elimination of gluten and/or wheat from their diets. One prospective study looking at the effects of a wheat-free diet on symptoms in 200 patients with NCGWS found that subjects who adhered strictly to a GFD had greater improvements in symptoms than those who did not (98% vs. 58%) [17]. However, this study had no control arm and did not assess the effects on sleep.

Unlike CD, little is known about the duration and extent to which patients should adhere to a GFD. There are no clear guidelines with respect to this or what benefits it would offer [6,18]. Adhering to a GFD is a significant undertaking; it is more costly and restrictive than a gluten-based diet. Adherence to a GFD for patients with CD is challenging and varies considerably, ranging from 42 to 91% [19,20]. It is therefore important to establish the benefits of adhering to a GFD for patients with NCGWS to allow for appropriate consultation and reduce these financial and nutritional challenges. 

At present, there is little known about the current rates of adherence to a GFD in this group [1,21]. This study aimed to compare rates of adherence to a GFD in patients with CD and NCGWS. We then sought to establish the extent to which adherence to a GFD impacts quality of life (QoL) and sleep performance in patients with NCGWS. As outlined, NCGWS is a complex condition that may encompass a range of sensitivities with challenging diagnostic criteria, and little is known about the long-term treatment. We hope this study will provide valuable data about adherence rates in NCGWS and how they compare to CD, as well as the affects adherence to a GFD has on QoL and sleep performance, to better guide our understanding of how to treat this condition. 

## 2. Methods

A prospective observational study was undertaken at Sheffield Teaching Hospitals, a specialist tertiary centre that manages patients with CD and other gluten-related disorders. Two cohorts of patients were identified for this study: one with NCGWS, and the other with CD. All patients were aged 18 and over.

### 2.1. NCGWS Cohort

All NCGWS patients were identified from a specialist gluten-related disorders clinic between February and July 2018, having been referred with self-reported gluten sensitivity using the cohort first described by Croall et al. [22]. All patients had to have negative serological tests (IgA-ttg and IgA-EMA), normal duodenal biopsies and reported improvement in their symptoms upon initiation of a gluten-free diet. Following exclusion of CD and other possible diagnoses, a clinical diagnosis of NCGWS was made and these patients enrolled. 

Patients then completed three validated assessment tools as part of routine clinical practise to assess their symptoms: the Coeliac Disease Assuagement Test (CDAT), the Coeliac Symptom Index (CSI) and the Sleep Condition Index (SCI). These tools were used to assess adherence to a GFD, QoL and sleep performance, respectively. Whilst the CDAT and CSI were designed and validated for use in CD, they were deemed suitable for use in patients with NCGWS given the large overlap between the two conditions. They were modified where appropriate to ensure they were applicable to NCGWS. 

The CDAT is a validated tool used to assess levels of adherence to a GFD in patients with Coeliac Disease. It is a questionnaire that consists of seven questions on a five-point Likert scale, with an overall numerical score ranging from 7 to 35: <8 points—excellent adherence, 8–12 points—very good adherence, 13–17 points—insufficient/inadequate adherence and >17 points—poor adherence [23,24]. 

The CSI is a multiple-choice questionnaire that allows for the disease-specific monitoring of symptoms. It asks questions about the patients’ symptoms and elements of their general health that are known to be relevant to CD. Scores range from 16 to 80. A score of 30 or less is associated with a high quality of life and excellent adherence to a GFD, whereas a score of 45 or more is associated with a poor quality of life and worse GFD adherence [25].

The SCI is an eight-item multiple-choice questionnaire validated for the assessment of sleep quality. Scores range from 0 to 32, with a higher score indicating better sleep. It appraises insomnia symptoms against *Diagnostic and Statistical Manual of Mental Health Disorders Volume Five* (DSM-V) criteria [26,27]. 

The results of these assessment tools were used to quantify rates of adherence to a GFD and correlation to QoL and sleep performance. 

### 2.2. CD Cohort

A second group of patients, with a biopsy-confirmed diagnosis of CD, were prospectively identified between March 2013 and December 2019 from the coeliac specialist clinic. This group completed the unmodified CDAT questionnaire only. The results were compared with the CDAT responses from the NCGWS group. 

### 2.3. Statistical Analysis

Statistical analysis was performed using GraphPad Prism 8.0 (GraphPad Software, Inc., San Diego, CA, USA). Continuous variables were compared using the Mann–Whitney U test, while categorical variables were compared using the Chi-square test or the Fisher’s exact test. *p* values were two-tailed, with a value of <0.05 representing statistical significance. A Spearman rank correlation coefficient was computed to assess the relationship between CSI/SCI and CDAT scores.

## 3. Results

### 3.1. NCGWS Cohort

In total, 125 patients were included, of which 114 patients had fully completed the CDAT questionnaires to allow a CDAT score to be calculated. For the CSI and SCI questionnaires, a total of 113 and 110 responses were recorded, respectively. Patients were predominantly female (84.8%) with a median age of 46 years (IQR: 35–59). 

The median CDAT score for patients with NCGWS was 17.0 (IQR: 13.25–20), with 37.7% (43/114) demonstrating inadequate adherence, and 43.0% (49/114) demonstrating poor adherence. Only 19.3% (22/114) of patients showed good adherence, and no patients scored <8 points, which would indicate excellent adherence. There was no significant difference in adherence between genders (*p* = 0.48). 

The median CSI score was 44 (IQR: 37–52). Using the recommended cut-off for this tool, 39.8% (45/113) of patients had a poor quality of life, whilst only 13.3% (15/113) had a high quality of life.

The overall median SCI score was 14.0 (IQR: 9–22), and the proportion of participants who met DSM-V criteria for insomnia disorder was 41.8% (46/110). 

**Figure 1:**
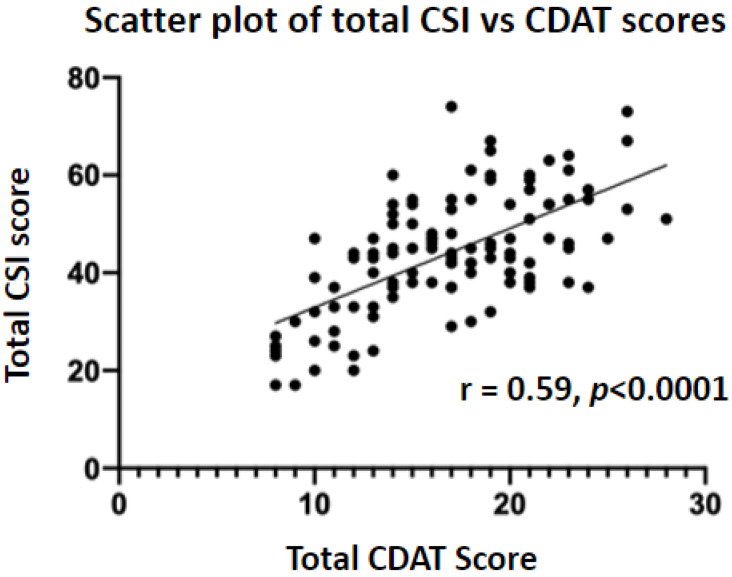
A scatter plot showing a positive correlation between CDAT and CSI scores. This indicates that poor GFD adherence (higher CDAT score) is associated with a lower quality of life (higher CSI score).

**Figure 2:**
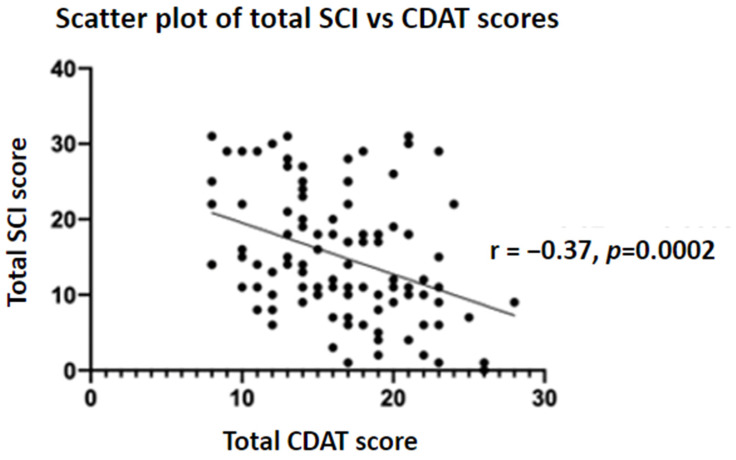
A scatter plot showing a negative correlation between CDAT and SCI scores. This indicates that poor GFD adherence (higher CDAT score) is associated with worse sleep performance (lower SCI score).

There was a positive correlation between total CSI and CDAT scores (r = 0.59, *p* < 0.0001). SCI and CDAT scores were found to be moderately negatively correlated (r = −0.37, *p* = 0.0002) (Figure 1 and Figure 2). 

### 3.2. CD Cohort

A total of 170 responses was recorded for patients in the CD cohort. There was a female predominance (71.2%), with a median age of participants of 52 years (IQR: 37.25–61.75). 

The median CDAT score for patients with CD was 13.0 (IQR: 10–15), with 37.6% (64/170) demonstrating inadequate adherence, and 13.5% (23/170) demonstrating poor adherence. Furthermore, 44.7% (76/170) reported good adherence, and 4.1% (7/170) scored <8, indicating excellent adherence. 

The median time that patients reported using a GFD was 63 months. There was no correlation observed between duration of disease and adherence (Spearman r = 0.10, *p* = 0.18). 

### 3.3. Comparing Adherence between the NCGWS and CD Cohort

There was a greater proportion of females (84.8%) in the NCGWS cohort than in the CD cohort (71.2%, *p* = 0.0039). There was no significant difference between the age of participants in both groups (*p* = 0.12). 

Patients with NCGWS had a higher median CDAT score, representing lower rates of adherence, when compared with the CD group (17.0 vs. 13.0, respectively; *p* = 0.0001). A greater proportion of patients with CD (83/170, 48.8%) reported good or excellent adherence compared with NCGWS (22/114, 19.3%, *p* < 0.0001).

A greater proportion of females with CD demonstrated good or excellent adherence to a GFD compared to those with NCGWS (48.8% vs. 18.2%, respectively; *p* < 0.0001). Conversely, there was no significant difference in male patients with CD or NCGWS who had good or excellent adherence (49.0% vs. 27.7%, respectively; *p* = 0.15). 

## 4. Discussion

To our knowledge, this study provides the largest comparison of adherence to a GFD in patients with CD and self-reported NCGWS. A previous study used the Salerno criteria to identify 44 patients with NCGWS and followed them up over at least one year on a GFD. They found that, similar to our study, patients had a significant symptom burden when not adhering to a gluten-free diet, and that adhering to a GFD significantly reduced these symptoms [28]. Whilst the Salerno criteria represent the expert consensus for the diagnosis of NCGWS, in clinical practice this can be difficult, and therefore we present real-world clinical data on patients with self-reported NCGWS. We found that patients with NCGWS adhered to a GFD less often than those with CD. Furthermore, lower rates of adherence to a GFD in patients with NCGWS were associated with a poorer QoL and worse sleep performance.

There are several factors that might explain why adherence was found to be better in the CD group. CD is a well-recognised condition by both the public and clinicians, whereas awareness of NCGWS is limited, and in the UK healthcare system it receives less support. This greater awareness is known to improve adherence [29]. Patients with CD often receive support from healthcare services, in particular dietetic support and advice pertaining to going gluten-free, whereas patients with NCGWS are often left to guide themselves through the challenging task of eliminating or reducing their gluten intake. Increasingly, many patients with CD engage with patient advocacy groups, such as Coeliac UK, allowing patients to share and receive information, which can contribute to better adherence [30], yet there is little support at present for patients with NCGWS. It has been shown that patients who are given individualised advice about gluten-containing foods and the GFD from healthcare professionals have better adherence to a GFD [31]. The role of follow-up and monitoring of patients in NCGWS is unclear; however, these patients may benefit from ongoing dietetic support to individualise the approach to those with NCGWS and support appropriate education to minimise the risks associated with a GFD. Committing to a GFD can be a difficult undertaking, with numerous barriers including cost (gluten-free products cost 159% more than regular items [32]), concerns about travelling or dining out, limitations of food choices and the challenge of continuing a GFD in moments of low mood or stress [30]. This highlights further why support and guidance from healthcare professionals can be beneficial. 

Previous studies reporting GFD adherence in individuals with NCGWS have shown mixed results. Interestingly, a study of 24 Mexican patients reported adherence to a GFD amongst patients with NCGWS was higher than those with CD [21]. A further study of 34 patients with NCGWS from Norway found no significant difference in adherence between the two groups, suggesting there may be cultural differences in adherence [33]. However, neither study used a validated method to measure adherence nor used a comparison to patients with biopsy-confirmed CD; they also had smaller sample sizes than our study. 

It is important that clinicians be aware of both the challenges and potential benefits when discussing the GFD with a patient with NCGWS. The role of a GFD in NCGWS is less clear than in CD; however, our data provides evidence of symptomatic benefits that should be considered depending on a patient’s presentation [1,34]. This finding was corroborated by a prospective study carried out by Carroccio et al. that showed patients who adhered strictly to a wheat-free diet had greater improvements in symptoms than those who did not, using the IBS Global Assessment of Improvement (GAI) tool [17]. However, NCGWS is a complex condition and may encompass multiple food-group sensitivities [13]. This makes diagnosis challenging and may limit the number of patients who are appropriately diagnosed and therefore treated. Furthermore, there is significant heterogeneity in the way patients with NCGWS present, with one study reporting that only 16% of patients with NCGWS demonstrated gluten-specific symptoms following a gluten challenge, and up to 40% of patients had a nocebo response [35]. The challenges of diagnosing a patient with NCGWS may mean that patients who could benefit from a GFD do not have the option discussed [8]. Therefore, whilst there is evidence to support clinicians in recommending a GFD to patients with NCGWS, it is not as comprehensive or clear-cut as it is for CD, which may diminish rates of adherence in this group [35]. 

When examining the rates of adherence of patients with CD, less than half (48.8%) reported good or excellent adherence. Whilst this was significantly higher than the patients with NCGWS (19.3%), it remains at the lower end of the reported literature (42–91%) [19,20]. This may represent the barriers to following a GFD discussed earlier. 

The CSI scores in this study offer valuable information about the QoL for patients with NCGWS. Only 13.3% of patients met the criteria for having a high QoL whilst 39.4% of patients scored in the range associated with a poor QoL. When these results are correlated with the CDAT scores, there is a statistically significant positive correlation between them (r = 0.59, *p* < 0.0001), with a higher CDAT score (indicating worse adherence) correlating with a higher CSI score (associated with a worse QoL). Whilst we were unable to make this comparison to the CD group, it has been corroborated by Leffler et al. (2009) [25]. Similar to our findings in patients with NCGWS, they found that a higher CSI score was associated with a poor QoL and worse GFD adherence in patients with CD. Our findings provide a valuable link between adherence and QoL for patients with NCGWS and reaffirms the role of a GFD in the treatment of NCGWS. 

When examining sleep performance in the NCGWS group, a significant proportion (41.8%) met the DSM-V criteria for insomnia disorder, according to the SCI results. A similar trend has been found in patients with CD. A nationwide study looking at sleep performance found that patients with CD have an increased risk of poor sleep, both before and after diagnosis [36]. Sleep is an important factor that contributes to QoL and can contribute to depression, anxiety and fatigue [37]. Zingone et al. (2010) found that patients with CD had higher Pittsburgh Sleep Quality Index (PSQI) scores than healthy volunteers, indicating worse sleep performance in the CD group [37]. Moreover, these were inversely linked to QoL scores. Interestingly, adherence to a GFD did not improve PSQI scores in their study; however, they compared patients with a new diagnosis of CD to those with ‘treated CD’ without quantifying the degree of adherence to a GFD and did not use a validated tool to assess adherence. This is in contrast to our findings, in which patients with NCGWS had a statistically significant negative correlation between CDAT and SCI scores, indicating poor GFD adherence is associated with worse sleep performance.

A limitation of this study is that we were unable to compare CSI and SCI scores for patients with NCGWS to patients with CD. There are several studies in the literature that have looked at QoL and sleep in patients with CD [25,36,37]. Nonetheless, we present the largest comparison of adherence between these groups, giving valuable insight into the two conditions. Another limitation is that we measured adherence through the use of questionnaires alone. The literature supports the use of multiple assessment tools in measuring adherence [31]. However, in clinical practice, this can prove challenging, and therefore we offer the questionnaire as a quick assessment that provides immediate feedback during the consultation. Assessing adherence to a GFD for patients with NCGWS can be challenging, as there is no validated questionnaire to assess this specifically for NCGWS. The CDAT questionnaire is validated in CD [23,24] and therefore seemed an appropriate tool to use in patients with NCGWS. We modified it where appropriate to ensure it was applicable to NCGWS. 

It should be noted that the consequences of not adopting a GFD in patients with CD can be significant, and the complications of CD are well recognised in the literature [38]. This means that patients with CD need strict adherence to a GFD. On the other hand, patients with NCGWS are often advised to maintain the maximum tolerated dose of gluten in their diet [39]. Therefore, making a direct comparison between these two groups can be misleading. However, our data show that patients on a GFD with NCGWS have a better QoL, and therefore perhaps a stricter GFD should be recommended to these patients with regards to their symptoms. 

In conclusion, patients with self-reported NCGWS had lower adherence rates to a GFD compared with patients with CD. Better adherence to a GFD was associated with higher QoL and sleep quality scores in patients with NCGWS. This reinforces that a GFD should be discussed as a treatment option in patients with NCGWS. Further work is needed to establish whether lifelong strict adherence is required, as is the case for CD, to prevent possible complications. Given that our findings suggest that adherence to a GFD is generally poor in this group and significantly lower than for patients with CD, work is needed to target this group of patients with dietetic and specialist support to understand why adherence is poor and to explore ways to improve it.

## Data Availability

Not applicable.
